# Inhibition of Src homology 2 domain‐containing phosphatase 1 increases insulin sensitivity in high‐fat diet‐induced insulin‐resistant mice

**DOI:** 10.1002/2211-5463.12000

**Published:** 2016-01-04

**Authors:** Janine Krüger, Ernst Wellnhofer, Heike Meyborg, Philipp Stawowy, Arne Östman, Ulrich Kintscher, Kai Kappert

**Affiliations:** ^1^ Center for Cardiovascular Research/CCRInstitute of Laboratory MedicineClinical Chemistry and PathobiochemistryCharité – UniversitätsmedizinGermany; ^2^ Department of Medicine/CardiologyDeutsches HerzzentrumGermany; ^3^ Cancer Center KarolinskaKarolinska InstitutetStockholmSweden; ^4^ Center for Cardiovascular Research/CCRInstitute of PharmacologyCharité – Universitätsmedizin BerlinGermany

**Keywords:** bis(maltolato)oxovanadium(IV), insulin resistance, protein tyrosine phosphatases, PTP inhibition, SHP‐1/Ptpn6, sodium stibogluconate

## Abstract

Insulin resistance plays a crucial role in the development of type 2 diabetes. Insulin receptor signalling is antagonized and tightly controlled by protein tyrosine phosphatases (PTPs). However, the precise role of the PTP src homology 2 domain‐containing phosphatase 1 (SHP‐1) in insulin resistance has not been explored. Male C57BL/6J mice were fed a high‐fat diet (HFD, 60% kcal from fat), to induce insulin resistance, or a low‐fat diet (LFD, 10% kcal from fat) for 10 weeks. Afterwards, HFD‐fed mice were pharmacologically treated with the SHP‐1 (Ptpn6) inhibitor sodium stibogluconate and the broad spectrum pan‐PTP inhibitor bis(maltolato)oxovanadium(IV) (BMOV). Both inhibitors ameliorated the metabolic phenotype, as evidenced by reduced body weight, improved insulin sensitivity and glucose tolerance, which was not due to altered PTP gene expression. In parallel, phosphorylation of the insulin receptor and of the insulin signalling key intermediate Akt was enhanced, and both PTP inhibitors and siRNA‐mediated SHP‐1 downregulation resulted in an increased glucose uptake *in vitro*. Finally, recombinant SHP‐1 was capable of dephosphorylating the ligand‐induced tyrosine‐phosphorylated insulin receptor. These results indicate a central role of SHP‐1 in insulin signalling during obesity, and SHP‐1 inhibition as a potential therapeutic approach in metabolic diseases.

**Abbreviations**
BMOV: bis(maltolato)oxovanadium(IV)
GTT: glucose tolerance test
HFD: high‐fat diet
ITT: insulin tolerance test
LFD: low‐fat diet
PTP: protein tyrosine phosphatase(s)
SHP‐1: src homology 2 domain‐containing phosphatase 1

Receptor tyrosine kinases (RTKs), like the insulin receptor, are tightly controlled by protein tyrosine phosphatases (PTPs), which together regulate the network cellular tyrosine‐phosphorylation status 1, 2, 3, 4. Insulin initiates a signalling cascade after ligation of the insulin receptor, leading to phosphorylation of the insulin receptor itself, substrates, and downstream signalling components 5, which is antagonized by different PTPs. Imbalance of insulin signalling components can lead to impaired glucose utilization and hyperglycaemia, as being evident in diabetes and insulin resistance 6, 7.

Clinically, insulin resistance is characterized by reduced systemic insulin‐response. Besides other causes, obesity has been found being both associated with and promoting of insulin resistance. Strikingly, in insulin resistance upregulation and altered activity of PTPs have been detected 8, 9, 10.

While 38 classical PTPs with sole tyrosine‐dephosphorylation activity are encoded in the human genome 11, only a limited number of PTPs was shown to target the insulin receptor 12. PTP1B, DEP‐1, LAR and TC‐PTP are known to dephosphorylate tyrosine‐phosphorylated residues of the insulin receptor 13, 14, 15, 16, 17, while SHP‐2 was shown to function as a positive regulator of insulin action 18. Further, Src homology region 2 domain‐containing phosphatase‐1 (SHP‐1) impacts on insulin signalling 19, 20. SHP‐1‐deficient mice (*Ptpn6* ^me−v/me−v^, also known as *viable motheaten*) were characterized by enhanced insulin receptor signalling in skeletal muscle and liver 19. Moreover, in experimental diet‐induced obesity SHP‐1 was shown to be upregulated in the metabolically active tissues skeletal muscle, adipose tissue and the liver 8.

Sodium stibogluconate represents a SHP‐1 inhibitor, which has also been used clinically in the treatment of leishmaniosis 21. Using sodium stibogluconate we tested the hypothesis that SHP‐1 inhibition leads to improvement of insulin sensitivity in a mouse model of high‐fat diet (HFD) induced insulin resistance. These analyses were extended by analyses of the impact of the broad spectrum pan‐PTP inhibitor bis(maltolato)oxovanadium(IV) (BMOV).

## Results

### Increased insulin signalling with sodium stibogluconate

**Figure 1:**
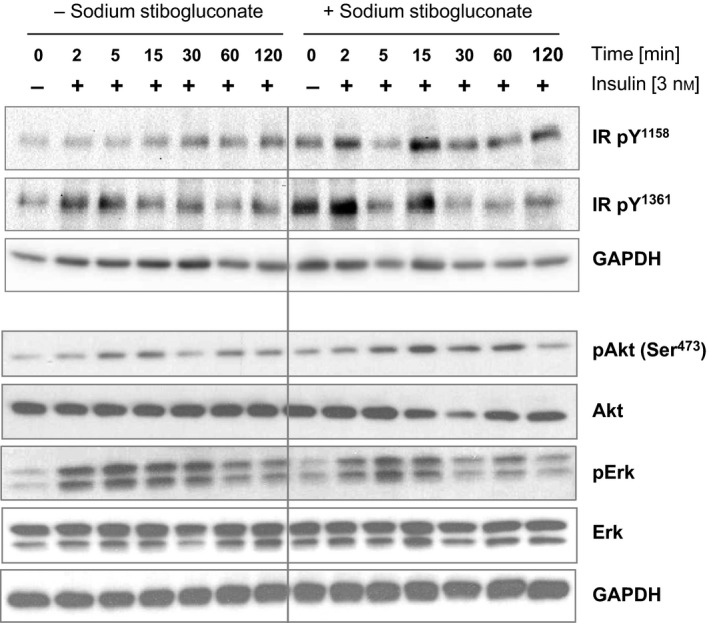
SHP‐1 inhibition with sodium stibogluconate increased insulin receptor phosphorylation. AML12 cells were treated with or without sodium stibogluconate followed by insulin stimulation [3 nm] for immunoblotting. Site‐specific phosphorylation levels of the insulin receptor and insulin signalling downstream kinases Akt and Erk were analysed. Shown is one representative immunoblot of *n* = 3 independent experiments.

Sodium stibogluconate, an effective SHP‐1 inhibitor 22, was used in the liver cell line AML12 to assess the inhibitory impact of SHP‐1 in insulin signalling *in vitro*. Immunoblotting analyses were performed to assess the site‐specific tyrosine phosphorylation of the insulin receptor and phosphorylation of downstream kinases Akt and Erk1/2 (Fig. 1). Insulin receptor phosphorylation at sites Y^1158^ and Y^1361^ was enhanced after SHP‐1 inhibition both under basal conditions and after insulin incubation. Particularly, tyrosine phosphorylation at site Y^1158^ was enhanced after 15 min, whereas the tyrosine site Y^1361^ revealed an increased phosphorylation already after short time (2 min) insulin stimulation. Further, the downstream kinase Akt was also activated after sodium stibogluconate treatment, while Erk1/2 was not affected. Specifically, Akt phosphorylation at Ser^473^, a commonly used measure for insulin sensitivity 8, 9, 23, was increased after long term insulin stimulation in the presence of sodium stibogluconate, while insulin‐induced Erk phosphorylation remained unchanged.

Together, sodium stibogluconate treatment resulted in a specific SHP‐1 inhibition associated with increased phosphorylation of the insulin receptor and Akt at defined times.

### HFD in C57BL/6J mice induced insulin resistance

**Figure 2:**
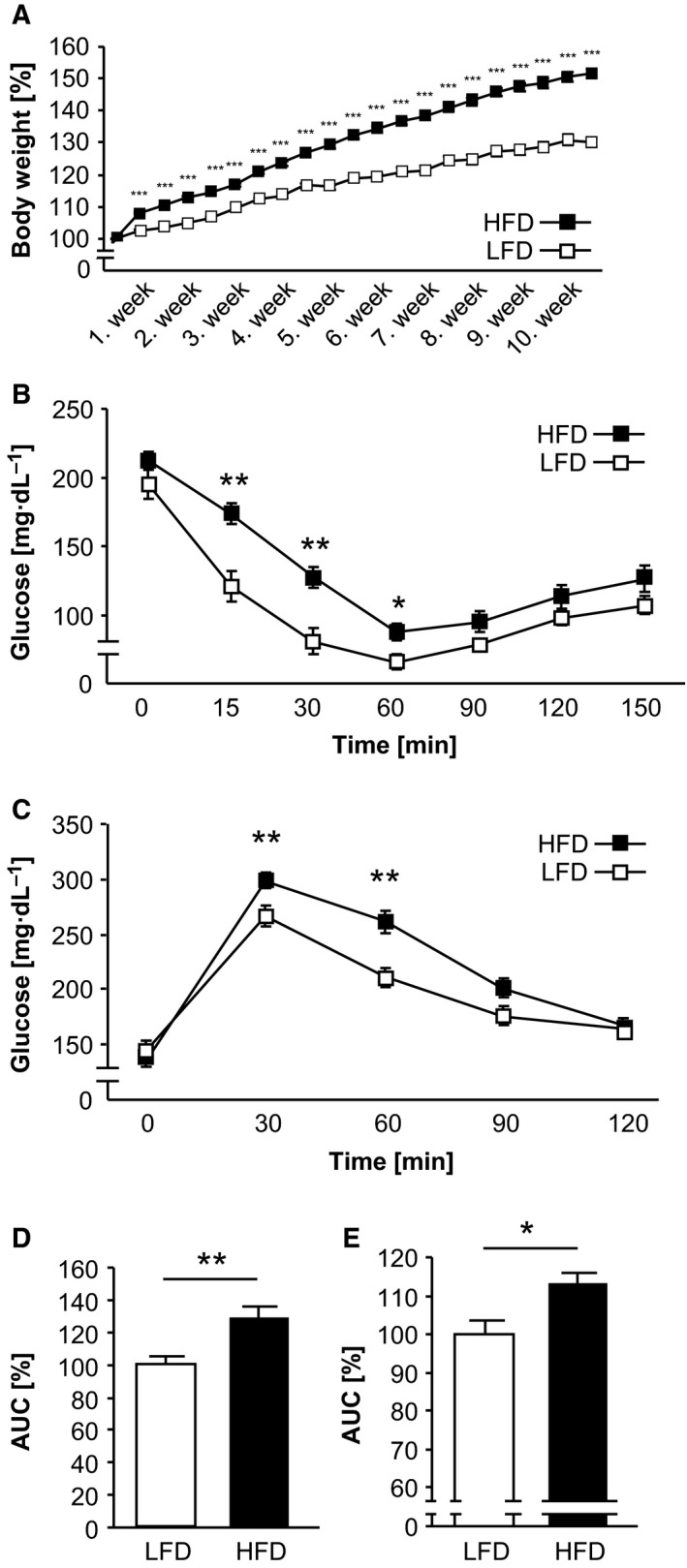
HFD feeding induced insulin resistance in C57BL/6J mice. Mice were fed a LFD and HFD over a period of 10 weeks. (A) The body weight was determined twice weekly. (B–E) Metabolic phenotyping included an ITT (B) and GTT (C) in fasted mice and the AUC was calculated for ITT (D) and GTT (E). *P < 0.05; **P < 0.01; ***P < 0.001 LFD vs. HFD; LFD:* n* = 10; HFD:* n* = 15.

HFD mice were characterized by a significantly higher weight gain during the first 10 weeks compared to LFD mice (Fig. 2A). Insulin resistance was induced only in mice subjected to an HFD, whereas control mice fed an LFD remained insulin sensitive, assessed by an insulin tolerance test (ITT) (Fig. 2B). Glucose concentration was significantly increased in HFD‐fed mice in the early period after insulin stimulation (15 min, 30 min and 60 min), in contrast to LFD mice. In addition, glucose tolerance was also reduced in mice under HFD, based on significantly increased blood glucose concentration after 30 min and 60 min in a glucose tolerance test (GTT) (Fig. 2C). Calculation of the area under the curve (AUC) for ITT and GTT confirmed the significantly reduced insulin sensitivity (Fig. 2D) and significantly impaired glucose tolerance (Fig. 2E) in HFD mice.

Thus, HFD feeding resulted in both impaired glucose and insulin tolerance.

### PTP inhibition with sodium stibogluconate and BMOV improved the metabolic phenotype in HFD‐induced insulin‐resistant mice

**Figure 3:**
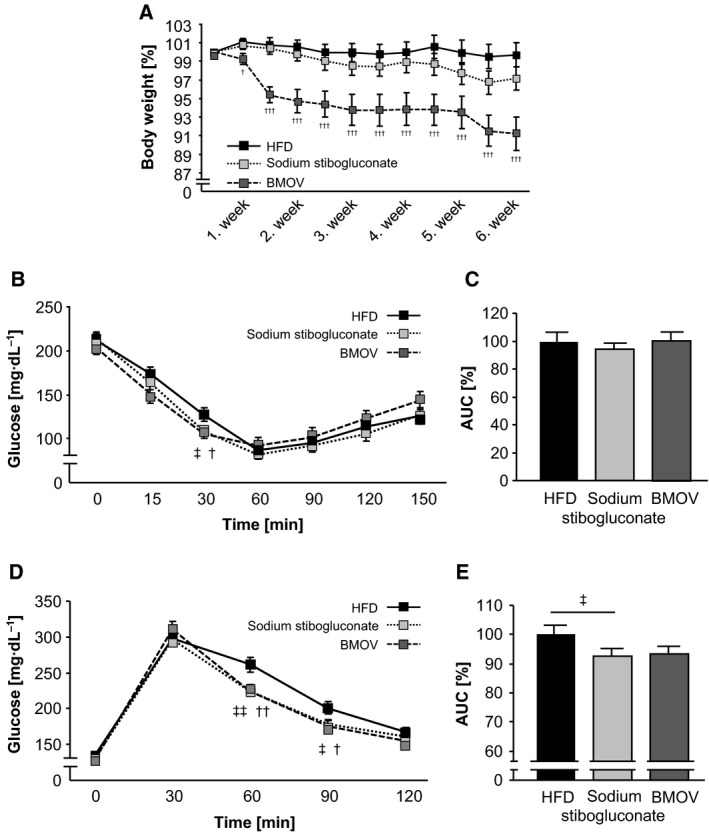
Pharmacological PTP inhibition increased insulin sensitivity and glucose tolerance in HFD mice. (A) Body weight was measured twice weekly during the pharmacological treatment. (B) An ITT was performed after PTP inhibition in fasted mice. (C) The AUC was calculated for ITT. (D) A GTT was carried out in all treatment groups with fasted mice. (E) The AUC was calculated for GTT (E). ^‡^ *P* < 0.05; ^‡‡^ *P* < 0.01 sodium stibogluconate vs. HFD; ^†^ *P* < 0.05, ^††^ *P* < 0.01, ^†††^ *P* < 0.001 BMOV vs. HFD; HFD, sodium stibogluconate, BMOV: each group *n* = 15.

To investigate whether PTP inhibition led to an improved metabolic phenotype after induction of insulin resistance, HFD mice were pharmacologically treated with the specific SHP‐1 inhibitor sodium stibogluconate or the broad spectrum pan‐PTP inhibitor BMOV for 6 weeks. HFD mice exhibited a constant body weight during this period, while mice treated with sodium stibogluconate showed a slight but insignificant decrease compared to HFD mice. In contrast, BMOV treatment resulted in a significant reduction of body weight (Fig. 3A).

Further, the metabolic status was assessed by ITT and GTT measurements. After insulin injection the glucose concentration decreased in both sodium stibogluconate and BMOV treatment groups, and achieved statistical significance after 30 min compared to HFD mice (Fig. 3B). However, the improved insulin sensitivity in the early period in sodium stibogluconate and BMOV‐treated mice did not translate into a significantly decreased AUC, in particular due to slightly higher glucose values at the late phase (Fig. 3C). Furthermore, glucose injection resulted in reduced blood glucose concentration in both sodium stibogluconate and BMOV groups with significantly decreased blood glucose values after 60 min and 90 min compared to HFD mice (Fig. 3D). This was consistent with a significantly reduced AUC in mice treated with sodium stibogluconate (Fig. 3E). However, the change in the AUC in BMOV‐treated animals did not reach statistical significance, based on minor differences in glucose levels at the 30 min time point.

**Figure 4:**
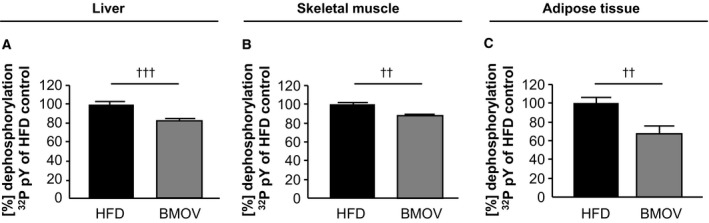
BMOV treatment reduced pan‐PTP activity in metabolic tissues. Measurements of PTP activity were performed in liver (A), skeletal muscle (B) and adipose tissue (C) of BMOV treated mice compared to HFD mice. Crude protein lysates were used for activity measurement with a radioactive labelled peptide. ^††^ *P* < 0.01 HFD vs. BMOV; ^†††^ *P* < 0.001 HFD vs. BMOV; *n* = 10–11 for each group.

To confirm the efficacy of the pharmacological treatment with BMOV in insulin‐resistant mice, metabolic tissues were used for pan‐PTP activity measurements. Liver, skeletal muscle, and adipose tissue revealed a significantly reduced PTP activity in all analysed tissues compared to HFD mice (Fig. 4A–C).

**Figure 5:**
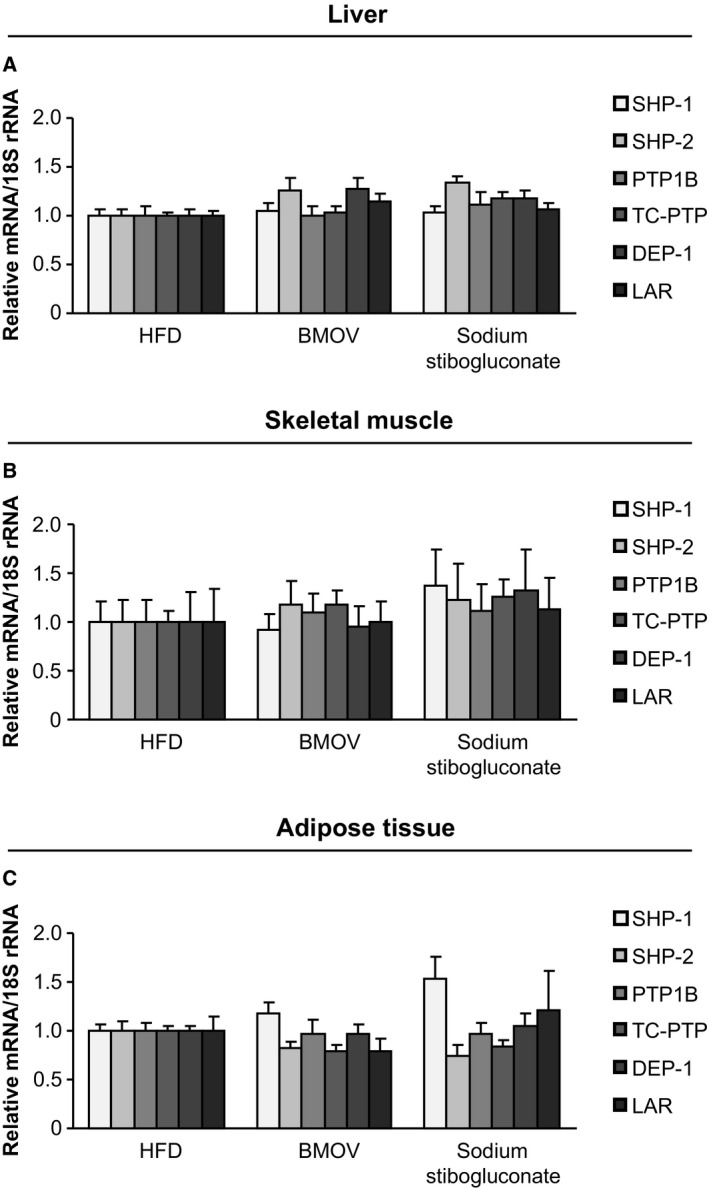
PTPs are not differentially expressed after PTP inhibition in metabolic tissues. Measurements of PTP expression on mRNA level were performed in liver (A), skeletal muscle (B) and adipose tissue (C) of BMOV and sodium stibogluconate treated mice compared to HFD mice.

In addition to PTP activity measurements, mRNA levels were analysed to rule out counterregulation of different PTPs caused by inhibition with BMOV and sodium stibogluconate in liver, skeletal muscle and adipose tissue compared to HFD mice. As shown in Fig. 5A–C, no significant differences in gene expression levels were detected for SHP‐1, SHP‐2, PTP1B, TC‐PTP, DEP‐1 and LAR in the three analysed animal groups.

To summarize, PTP inhibition with sodium stibogluconate and BMOV improved the metabolic phenotype in HFD mice, in particular with regard to glucose utilization.

### Glucose uptake is increased in C2C12 cells after PTP inhibition with sodium stibogluconate and BMOV

**Figure 6:**
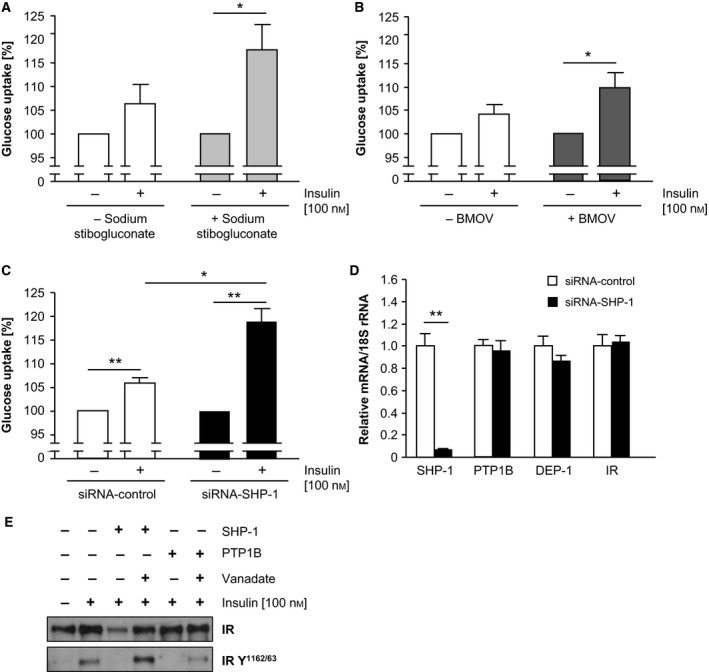
PTP inhibition resulted in enhanced glucose uptake accompanied by increased insulin receptor phosphorylation. (A‐B) Differentiated C2C12 cells were treated with sodium stibogluconate (A), BMOV (B) and transfected with SHP‐1 siRNA (C) followed by measurement of insulin‐induced glucose uptake. Expression of mRNA was analysed to confirm efficient SHP‐1 downregulation and to rule out compensatory regulation of the insulin receptor and other PTPs (D). (*P < 0.05; **P < 0.01). (E) Dephosphorylation assay was performed with precipitated insulin receptor, which served as substrate for recombinant SHP‐1 and PTP1B. To visualize the dephosphorylation of the insulin receptor immunoblotting was done with depicted antibodies.

PTP inhibition led to increased phosphorylation in insulin signalling *in vitro* accompanied by an improved glucose tolerance after PTP inhibition *in vivo*. Thus, to investigate whether muscle cells were impacting on the improved glucose homeostasis, differentiated C2C12 cells were used for glucose uptake assays. Insulin stimulated glucose uptake was examined without PTP inhibition and in sodium stibogluconate and BMOV‐treated cells. These results showed that PTP inhibition with both pharmacological compounds led to an increased insulin‐induced glucose uptake compared to untreated cells (Fig. 6A–B). To underline the impact of SHP‐1, additionally, siRNA‐mediated SHP‐1 downregulation was applied in C2C12 cells, followed by glucose uptake measurements. A significantly increased glucose uptake was observed after siRNA‐SHP‐1 transfection (Fig. 6C), consistent with significantly reduced SHP‐1 mRNA levels without counterregulatory changes in gene expression of the insulin receptor and other PTPs (Fig. 6D). Hence, PTP inhibition in skeletal muscle cells *in vitro* likely impacts on insulin signalling, demonstrated by improved glucose utilization. This is consistent with the ability of recombinant SHP‐1 protein to dephosphorylate the insulin‐induced phosphorylated insulin receptor (Fig. 6E). This capability of SHP‐1 for insulin receptor dephosphorylation was comparable to recombinant PTP1B. As PTP1B has earlier been described to dephosphorylate the insulin receptor at site Y^1162/63^ 24, we focused on analyses of this site. To confirm the tyrosine‐dephosphorylation activity, prior incubation with the PTP inhibitor sodium vanadate resulted in inactivation of SHP‐1 and PTP1B, leading to the loss of dephosphorylating capacity (Fig. 6E).

Taken together, PTP inhibition is strongly associated with increased insulin receptor phosphorylation, and sodium stibogluconate and BMOV treatment leads to significantly increased glucose uptake in skeletal muscle cells.

## Discussion

While SHP‐1 deficient mice are known to exhibit enhanced insulin signalling 8, 19, a pharmacological approach inhibiting SHP‐1 had not been performed previously. In this study, sodium stibogluconate, a specific SHP‐1 inhibitor, and BMOV, a broad spectrum pan‐PTP inhibitor were applied in HFD‐induced insulin‐resistant mice as a treatment approach. Both pharmacological regimens antagonized metabolic changes induced by HFD feeding. PTP inhibitor‐treated mice were characterized by increased insulin sensitivity and glucose tolerance *in vivo*, which was not due to altered PTP gene expression, including SHP‐1. Moreover, application of both PTP inhibitors showed an increased glucose uptake in skeletal muscle cells. This was consistent with enhanced phosphorylation of key intermediates in insulin signalling after SHP‐1 inhibition and glucose uptake after siRNA‐mediated SHP‐1 downregulation *in vitro*.

Deletion of serine/threonine phosphatases 25 and in particular protein tyrosine phosphatases 13, 14, 26 has earlier been shown to result in an improved metabolic phenotype in insulin resistance and diabetes. Moreover, elevated expression and activity of PTPs was detected in metabolic tissues in HFD‐induced insulin‐resistant mice earlier 8, 15, 27, including SHP‐1 8. Based on these data, SHP‐1 represents a potential therapeutic target for pharmacological intervention with sodium stibogluconate, a potent SHP‐1 inhibitor 22. Therefore, AML12 liver cells were chosen for analysing the phosphorylation level of intermediates of the insulin signalling pathway. After SHP‐1 inhibition, increased phosphorylation of the insulin receptor and the downstream kinase Akt, suggest that SHP‐1 may be a suitable drug target. This is consistent with the data recently received in mice with hepatocyte‐specific *Ptpn6* deletion, resulting in lower fasting glucose and improved hepatic insulin sensitivity 8. To analyse the impact of a pharmacological PTP inhibition *in vivo* a commonly used model of insulin resistance induced by HFD feeding was applied in mice 8, 9, 26, 28, 29. These animals were characterized by significant weight gain along with impaired insulin sensitivity and reduced glucose tolerance. Based on the *in vitro* data with enhanced insulin signalling in liver cells after SHP‐1 inhibition, insulin‐resistant mice were pharmacologically treated with sodium stibogluconate, also known to distribute *in vivo* 30. Furthermore, the impact of the broad spectrum pan‐PTP inhibitor BMOV, previously only analysed in diabetic rats 31, was explored. BMOV is characterized by a nonselective inhibition of different PTPs 32, including PTP1B 33. Metabolic phenotyping – body weight, ITT, GTT – revealed in both pharmacological groups a beneficially altered metabolic status in HFD‐induced insulin resistance. All analysed metabolic parameters were improved after PTP inhibition, evidenced by reduction in body weight, improved insulin sensitivity and enhanced glucose tolerance. Moreover, sodium stibogluconate and BMOV treatment – here for the first time in insulin‐resistant mice – showed a similar efficacy.

The metabolic phenotype could not be explained by differences in epididymal fat mass or physical activity (data not shown). In contrast, the improved glucose tolerance, measured by the GTT, was substantiated by increased glucose uptake obtained in mouse skeletal muscle cells after PTP inhibition and transfection of siRNA against SHP‐1 *in vitro*. Furthermore, data from rat L6 myocytes after adenoviral mediated expression of a catalytically inert DNSHP‐1 mutant 20 are consistent with our data obtained in mouse C2C12 cells. Recombinant SHP‐1, as the known component in insulin signalling PTP1B, was capable in insulin receptor dephosphorylation *in vitro*, which was antagonized by prior PTP inhibition. These data further support SHP‐1 as novel target for antidiabetic drugs.

BMOV is rapidly absorbed and distributed in various tissues 34. It has previously been reported to augment VEGF receptor and insulin receptor signalling and to modulate specific cell functions such as cell proliferation and insulin sensitivity in rats 31, 35. BMOV is an organic vanadate derivate with potent and broad spectrum PTP inhibition properties. Our observation that BMOV treatment resulted in a significant reduction in PTP activity in the metabolic tissues liver, skeletal muscle and adipose tissue validated adequate drug distribution *in vivo*. The precise underlying mechanism for BMOV‐induced weight loss remains to be determined. It might, at least partly, be due to altered insulin signalling based on PTP1B‐inhibitory action, since BMOV also inhibits PTP1B. In fact, PTP1B knockout has earlier been shown to protect against weight gain 13. Finally, our data are in accordance with weight loss in BMOV‐treated rats 36.

Sodium stibogluconate is primarily applied to treat leishmaniose infections 37 but it is also been used in a clinical phase I trial as an anticancer drug to target SHP‐1 38. Pharmadynamic studies with sodium stibogluconate earlier revealed drug efficacy in the liver 30. The inhibitory effect with recombinant SHP‐1 has been shown earlier 22. Furthermore, both pharmacological approaches did not influence the expression of the PTPs SHP‐1, SHP‐2, PTP1B, TC‐PTP, DEP‐1 and LAR, which all have been implicated as regulators in insulin signalling or insulin resistance earlier. Therefore, these data underline that the observed effects during 6 weeks BMOV and sodium stibogluconate treatment were due to inhibition of PTP activity and not due to expression changes. However, after *in vivo* application of sodium stibogluconate, given at a dose that was shown to exhibit significant antileishmanial property in rodents 39, we were not able to detect a strong SHP‐1 inhibition in isolated metabolic tissues (not shown), which is, however, consistent with previous observations 40. Therefore, we further performed sodium stibogluconate treatment of cultured AML12 cells, followed by immunoprecipitation of SHP‐1 and activity measurements. In these experiments we could detect a small reduction (~ 13%, *P* = 0.065) in SHP‐1 activity (not shown). This suggests that – contrasting the strong *in vivo* metabolic effects and impact on recombinant SHP‐1 22 – the lack of a significant detectable SHP‐1 inhibition in tissues and cultured cells is presumably based on either transient inhibitory efficiency of sodium stibogluconate *in vivo* or on processing specifics of the animal tissues *in vitro*. Furthermore, our data showing *in vivo* efficacy are underlined by demonstration of sodium stibogluconate clearly reducing leishmanial skin lesions in a clinical study in patients applying also the same drug concentration (20 mg·kg^−1^·day^−1^) as in our experimental protocol 41.

Inhibiting the ubiquitously expressed SHP‐1 in wild‐type mice clearly demonstrated the beneficial metabolic effects in our study, without causing any detectable side effects. However, the phenotype of two different conditional SHP‐1 knockout models should be acknowledged. *Motheaten* and *viable motheaten* mice, expressing no active or low levels of catalytic inactive SHP‐1, respectively, are characterized by severe abnormalities along with a reduced life span 42. Nevertheless, *viable motheaten* mice are also characterized by improved insulin sensitivity and glucose tolerance due to enhanced insulin signalling in liver and skeletal muscle.

Taken together, this study showed the therapeutic potential of sodium stibogluconate in metabolic diseases. Inhibiting SHP‐1 by sodium stibogluconate was followed by enhanced insulin signalling, leading to a phenotype in mice with increased insulin sensitivity and glucose tolerance. These data support a novel pharmacological approach to treat insulin resistance by sodium stibogluconate.

## Materials and methods

### Animals and treatment

C57BL/6J mice were purchased from Janvier (Le Genest‐Saint‐Isle, France). Mice aged 4–6 weeks were fed *ad libitum* a low‐fat diet (LFD) (*n* = 10) (10% kcal from fat; Altromin, Lage, Germany) or a high‐fat diet (HFD) (*n* = 45) (60% kcal from fat; Altromin) for 10 weeks. Afterwards, intraperitoneal application of sodium stibogluconate (Calbiochem, Schwalbach, Germany) [20 mg/kg body weight] (*n* = 15) was carried out daily, or twice weekly with BMOV (Organica, Wolfen, Germany) [0.75–3.0 mmol] in an escalating application scheme (*n* = 15), or daily of vehicle in 0.9% carboxymethylcellulose (*n* = 15) in HFD‐fed mice for additional 6 weeks as a treatment approach. After metabolic phenotyping mice were sacrificed under isoflurane anaesthesia. All animal procedures were in accordance with institutional guidelines and were approved by the Landesamt für Gesundheit und Soziales (LAGeSo, Berlin, Germany).

### Metabolic phenotyping (body weight, ITT, GTT)

Twice weekly body weight was recorded throughout the study period. In fasted mice an intraperitoneal insulin tolerance test (ITT) was performed by using insulin (Insuman^®^ Rapid, Sanofi Aventis, Berlin, Germany) in a dose of 0.5 U·kg^−1^ and an intraperitoneal glucose tolerance test (GTT) with 1 g·kg^−1^ glucose (Glucosteril, Fresenius, Bad Homburg, Germany). Glucose concentration of tail vein blood was measured at indicated time points by using a glucometer (Precision Xceed, Abbott, Wiesbaden, Germany).

### Cell culture, PTP inhibition glucose uptake and siRNA transfection

C2C12 myoblasts and AML12 liver cells were purchased from American Type Culture Collection (ATCC^®^, Wesel, Germany) and maintained in DMEM (Dulbecco‘s Modified Eagle Medium) or DMEM/F12, respectively, containing 10% FBS and 1% penicillin/streptomycin at 37 °C in an atmosphere of 95% air and 5% CO_2_. Insulin stimulation in AML‐12 was performed in cells fasted over night by adding insulin [3 nm] for indicated time periods. Differentiation of C2C12 cells to myotubes was carried out for 6 days followed by glucose uptake experiments as described earlier 14. PTP inhibition was done by adding sodium stibogluconate [11 μm] and BMOV [50 μm] – both dissolved in water – for 1 h before each experiment was performed. Transfection of C2C12 cells was carried out using 10 nm siRNA against SHP‐1 (Thermo Fisher Scientific, Bonn, Germany), and Lipofectamine^®^RNAiMAX (Invitrogen, Karlsruhe, Germany) for 72 h according to the manufacturer's (Invitrogen) recommendations. Cells transfected with nontargeting siRNA served as control.

### Protein tyrosine phosphatase activity

Pan‐PTP activity in metabolic tissues (liver, skeletal muscle, adipose tissue) was measured by using a radioactive labelled peptide as described previously 15.

### Quantitative real‐time PCR (qPCR)

RNA was isolated with RNeasy Mini Kit (Qiagen, Hilden, Germany) following the manufacturer's instruction for purification from C2C12 cells and tissue (liver, skeletal muscle, adipose tissue), and cDNA synthesis was done with SuperScript^®^II (Invitrogen). Quantitative real‐time PCRs were performed with SybrGreen (Applied Biosystems, Darmstadt, Germany) in duplicate per condition. The expression of analysed genes was normalized to the average expression of the housekeeping gene Rn18s.

The following primer sequences (final concentrations 100 nm) were used (forward and reverse respectively):

*Insr* 5′‐CAATGGGACCACTGTATGCATCT‐3′, 5′‐ACTCGTCCGGCACGTACAC‐3′;
*Ptpn1* 5′‐CGGGAGGTCAGGGACCTT‐3′, 5′‐GGGTCTTTCCTCTTGTCCATCA‐3′;
*Ptpn2* 5′‐GCTACGACGGCTCAGAAGGT‐3′, 5′‐TGTCTGTCAATCTTGGCCTTTTT‐5′;
*Ptprf* 5′‐ACCCGATGGCTGAGTACAAC‐3′, 5′‐GCCTTCACCTGTTTTGGGTA‐5′;
*Ptpn6* 5′‐CGTACCCTCCCGCTGTGA‐3′, 5′‐TTTTCGTACACCTCCTCCTTGTG‐3′;
*Ptpn11* 5′‐CCTCAACACAACTCGTATCAATGC‐3′, 5′‐TGTTGCTGGAGCGTCTCAAA‐3′;
*Ptprj* 5′‐GCAGTGTTTGGATGTATCTTT‐3′, 5′‐CTTCATTATTCTTGGCATCTGT‐3′;
*Rn18s* 5′‐GACTCTTTCGAGGCCCTGTA‐3′, 5′‐CACCAGACTTGCCCTCCAAT‐3′.

### Immunoblotting and dephosphorylation assay

Preparation of protein lysates and immunoblotting was done by standard protocols with primary antibodies (anti‐phospho insulin receptor Y^1158^, anti‐phospho insulin receptor Y^1361^, anti‐phospho insulin receptor Y^1162/63^ (Abcam, Cambridge, UK), anti‐phospho Akt (Ser^473^), anti‐pan Akt, anti‐insulin receptor (4B8), anti‐phospho Erk, anti‐pan Erk (Cell Signaling/New England Biolabs, Frankfurt, Germany) and anti‐GAPDH (Millipore, Schwalbach, Germany). The recombinant proteins PTP1B and SHP‐1 (Abcam) were used for the dephosphorylation assay with phosphorylated insulin receptor as described 15.

### Statistical analysis

Data are expressed as mean ± standard error of the mean (SEM). Statistically significant (*P* < 0.05) differences between the groups were determined by one‐way anova analysis with *post hoc* correction (Bonferroni) or *t*‐test and nonparametric tests (Mann–Whitney *U*) as appropriate (spss V 21, IBM, Ehningen, Germany).

## Author contributions

JK and KK designed and performed the study, performed experiments, analysed data, wrote, reviewed and edited the manuscript. EW analysed data and assisted with drafting the manuscript; HM performed experiments. PS, AÖ and UK critically revised the manuscript and contributed to interpretation and discussion. All authors approved the final version of the manuscript.
